# The Transcription Factor, α1ACT, Acts Through a MicroRNA Network to Regulate Neurogenesis and Cell Death During Neonatal Cerebellar Development

**DOI:** 10.1007/s12311-022-01431-2

**Published:** 2022-06-22

**Authors:** Cenfu Wei, Kellie Benzow, Michael D. Koob, Christopher M. Gomez, Xiaofei Du

**Affiliations:** 1 Department of Neurology, University of Chicago, Chicago, IL 60637 USA; 2 Department of Laboratory Medicine and Pathology, University of Minnesota, Minneapolis, MN 55455 USA

**Keywords:** α1ACT, CACNA1A, Cerebellum, Development, miRNA

## Abstract

MicroRNAs, a class of small RNA regulators, function throughout neurodevelopment, from neural stem cell neurogenesis to neuronal maturation, synaptic formation, and plasticity. α1ACT, a transcription factor (TF), plays a critical role in neonatal cerebellar development by regulating an ensemble of genes. Of these, ChIP-seq analysis matched near 50% genes directly regulated by α1ACT. Yet, more than half the regulated transcripts lacked direct interaction with α1ACT. To investigate whether α1ACT acts through a microRNA network, we studied α1ACT-associated simultaneous miRNA:mRNA transcriptome profiles, using miRNA-seq paired with RNA-seq. Thirty-one differentially expressed miRNAs (DEMs) associated with α1ACT-regulated differentially expressed genes (DEGs) were profiled in α1ACT-overexpressing PC12 cells and were further validated in neonatal transgenic mouse cerebellum overexpressing α1ACT in a context-dependent manner. Here, we also demonstrated that α1ACT facilitates neurogenesis and development of dendritic synapses and is partially a result of the downregulation of the miR-99 cluster, miR-143, miR-23, miR-146, miR-363, and miR-484. On the other hand, the miR-181, miR-125, and miR-708 clusters were upregulated by α1ACT, which inhibit MAPK signaling and cell death pathways by targeting Ask1, Odc1, Atf4, and Nuf2 for decreased expression. MiR-181a-5p was verified as the most abundant DEM in neonatal cerebellum, which was further induced by α1ACT. Overall, under α1ACT modulation, up-/downregulated miRNA clusters with their paired target genes may form a regulatory network controlling the balance between the neuronal proliferation, differentiation, and cell death in the cerebellum to promote neonatal development. Our findings concerning the α1ACT-related miRNA/mRNA expression profiles in neonatal cerebellum may inform future investigations for cerebellar development.

**Supplementary Information:**

The online version contains supplementary material available at 10.1007/s12311-022-01431-2.

## Introduction

The proliferation of neuronal precursor cells and the division of immature neurocytes are key to cerebellar morphogenesis during embryonic and fetal development [1, 2]. The neonatal cerebellum still retains considerable proliferative capacity [3–5] and is sustained by the activation of various transcriptional regulators [5–7] and endogenous microRNA(miRNA) mechanisms [8–11]. The maturation of the Purkinje cell (PC) dendritic tree and soma is also extended to early postnatal life, when cerebellar neurocytes still retain the ability to differentiate and proliferate [12–14]. However, the molecular mechanisms that induce differentiation and maturation of neonatal neurocytes toward cerebellar cells remain obscure.

MiRNAs are a class of short noncoding RNAs controlling the expression levels of their target genes at a post-transcriptional stage. At the molecular level, miRNAs exert their function by binding, with imperfect base pairing, to target sites on their specific mRNAs. This miRNA/mRNA binding either causes the inhibition of translational initiation or leads to mRNA degradation [15, 16]. Thus, to gain insight into the biological role of a single miRNA, it is essential to identify the full repertoire of its mRNA targets. The brain, which has a wide variety of neuronal and non-neuronal cell types, expresses larger number and a more distinct set of miRNAs than any other tissues [17]. The diversity and gene-regulatory capacity of miRNAs are particularly valuable in the neonatal cerebellum, where persistent functional specialization of neurons requires constant neuronal adaptation to environmental cues [18]. MicroRNAs that fine-tune protein synthesis have emerged as key factors in the regulation of brain and cerebellar development [19, 20].

We have shown that in cerebellar PCs, the expression of α1ACT, normally co-expressed with the α1A voltage-gated channel protein by the *CACNA1A* gene, is required for normal motor and cerebellar development [12, 21]. α1ACT, as a transcription factor, regulates a large ensemble of developmentally controlled genes, thereby orchestrating a network of pathways driving neurogenesis, synapse formation, and cell adhesion to mediate morphological and functional maturation of PCs and their connections. In previous studies, we demonstrated the mRNA expression profiles under the regulation of α1ACT, and showed that some of the mRNA expressions were under the regulation of α1ACT due to its genomic DNA binding patterns. In this study, we additionally correlated the mRNA expression profiling of α1ACT with miRNA as putative post-transcriptional regulators.

It has been suggested that the simultaneous expression profiling of miRNAs and mRNAs could be an effective strategy for miRNA target identification [22]. We performed a co-sequencing of miRNA and mRNA transcriptomes in pc12 cells with α1ACT overexpression and confirmed our target genes in cerebellar tissues. We demonstrated that miRNA and mRNA profiling is feasible to reveal regulatory mechanisms both upstream and downstream of intercellular miRNA heterogeneity and miRNA/mRNA interaction and identified several miRNAs as potential regulators.

## Materials and Methods

### Experiment Model and Subject Details

#### Ethics Statement

All animal experiments were approved and carried out in accordance with the regulations and guidelines for the care and use of experimental animals at the Institutional Animal Care and Use Committee at the University of Chicago.

#### α1ACT Overexpression Mouse Model

All mice were housed in groups of up to five littermates in special pathogen-free facility at the University of Chicago with a 12-h light/dark cycle. The BAC-α1ACT-3xFlag mice (C57BL/6j background) that carry a truncated human CACNA1A gene with 4 CAG repeats was generated by M Koob and K Kenzow at the University of Minnesota. A Purkinje cell-specific Tet-transactivator (tTA) driver construct was generated by fully replacing the coding region of the mouse Purkinje cell protein 2 (Pcp2, isoform 6) gene with the “tTA-Advanced” open reading frame (TaKaRa) in the mouse genomic BAC clone RP24-186D18 (BacPacResources). The α1ACT-3xFlag fragment driven by a Tet-responsive element (TRE) 3G promoter (TaKaRa) was inserted into the SP6 end of the BAC vector sequence in the same construct. This BAC construct was then integrated in single copy into the mouse genome in a nondisrupting site 3’ of the Col1A1 gene, as described [23]. C57BL6j mice were purchased from Jackson Laboratory.

##### ***Cell Lines***

Rat pheochromocytoma cell line pc12 was established from ATCC and cultured in F-12 K medium (ATCC, Manassas) supplemented with 10% horse serum and 2.5% fetal bovine serum (FBS) in a 37 °C, 5% CO2 incubator on a plate coated with 0.01% poly-L-lysine. For stable cell line generation, when pc12 cells reached 60% confluence, they were transfected with pcDNA3 vectors expressing C-terminal 3xFlag tag α1ACT with 11 polyglutamine repeats and empty pcDNA3 vector using TransIT 1(Mirus, Pittsburgh, PA, USA) by following the manufacturer’s instruction. Transfected cells were selected with 150 mg/ml G418(GIBCO), and then stable clones were obtained.

pc12 cells were derived from a male rat pheochromocytoma which was first cultured by Greene and Tischler in 1976 [24]. pc12 cells can be sub-cultured indefinitely and develop into neuronal phenotype in response to nerve growth factor. pc12 cells also can synthesize, store, and release neurotransmitters such as DA or noradrenaline [25] [26]. The pc12 cell line has been a classic model to study the physiology and pathology of the nervous system [27]. Studies have been shown pc12 cells are similar to the primary cultured neurons and have the features of mature dopaminergic neurons [28, 29] expressing ion channels and neurotransmitter receptor [30].

#### RNA Isolation and Quantitative Real-Time PCR

Total cellular RNA and total mouse brain and cerebellum RNA was isolated by RNeasy mini Kit and RNeasy lipid mini Kit (QIAGEN, Valencia, CA, USA), respectively, according to the manufacturer’s instructions. The flow-through obtained from the total RNA extraction was used for purifying the miRNA-enriched fraction by using the RNeasy MinElute Cleanup Kit (QIAGEN, Valencia, CA, USA), following the manufacturer’s instructions. miRNA adapter ligation, reverse-transcription, and amplification were proceeded using Taqman Advanced miRNA cDNA Synthesis Kit (Applied Biosystems, Waltham, MA) by following the manufacturer’s instructions. To conduct miRNA quantitative real-time PCR, each 10 μL reaction contained 0.5 μL Taqman advanced gene expression Assay (Thermal Fisher, Waltham, MA), 0.5 μL cDNA templates, 5 μl Taqman advanced gene expression Master Mix (Applied Biosystems, Waltham, MA), and 4 μL water and was ran under the following conditions: 95 °C 20 s; 95 °C 1 s, and 60 °C 30 s for 40 cycles. As a relative quantification, fold changes were measured using the ΔΔCt method, in which miR-186 and miR-192 were used as internal controls. To obtained mathematical significance, samples were triplicated, and each experiment was repeated at least three times.

mRNA cDNA was generated using SuperScript VILO cDNA Synthesis Kit (Thermal Fisher, Waltham, MA) following the manufacture’s instructions. To conduct mRNA quantitative real-time PCR, each 10 μL reaction contained 0.5 μL Taqman gene expression Assay (Thermal Fisher, Waltham, MA), 0.5 μL cDNA templates, 5 μl Taqman gene expression Master Mix (Applied Biosystems, Waltham, MA), and 4 μL water and was run under the following conditions: 50 °C 2 min; 95 °C 10 min, 95 °C 15 s, and 60 °C 60 s for 40 cycles. As a relative quantification, fold changes were measured using the ΔΔCt method, where GAPDH was used as an internal control. To obtain mathematical significance, samples were triplicated, and each experiment was repeated at least three times.

#### RNA and Small RNA NGS Sequencing

RNA-seq was described previously [12]. Small RNA-seq was performed using Illumina TrueSeq Small RNA protocol at the University of Chicago Genomics Facility.

#### Western Blot

Nuclear and cytoplasmic protein from cell and mouse cerebellum were extracted using NE-PER Nuclear and Cytoplasmic Extraction Reagents following the manufacturer’s protocol (Thermo Scientific, Waltham, MA). Protein concentration was tested using Bio-Rad protein assay (Bio-Rad, Hercules, CA). Twenty μg of total protein were subjected to SDS–PAGE (8%, 10% Tris–Glycine gel, Invitrogen, Grand Island, NY, USA) and transferred to a PVDF membrane (Millipore, Billerica, MA, USA). The transblotted membrane was blocked with TBS containing 5% non-fat milk and 0.05% Tween-20 for 60 min and then incubated with the primary antibody at 4 °C overnight. The membrane was then probed with HRP-conjugated secondary antibody for 1 h at room temperature and washed with TBS with 0.05% Tween20 three times. Finally, the immunoblots were detected using chemiluminescent substrate (Thermo Scientific Pierce Protein Biology Products, Rockford, IL, USA) and visualized by autoradiography.

#### Immunofluorescence

Immunohistochemistry was performed as previously reported [12, 21]. Briefly, paraffin-embedded sections of perfused brains were de-waxed and rehydrated and then steamed for 20 min in antigen retrieval solution (Reveal; Biocare Medical, Walnut Creek, CA, USA). Sections were blocked and exposed to primary antibody for 12 h at 4 °C. After washing, fluorescent secondary antibody in PBS-T (phosphate buffered saline and 0.05% Tween-20) was added for 1 h at room temperature. Confocal fluorescence microscopy was carried out under a Leica SP8 lighting confocal microscope (Leica, Buffalo Grove, IL, USA) at the University of Chicago Integrated Light Microscopy Core.

### Bioinformatical Analysis

#### miRNA-seq and Differential miRNA Expression Analysis

miRNA-seq reading data was proceeded by quality control using FastQC and adapter (TGGAATTCTCGGGTGCCAAGG) trimming using Cutadapter. The miRNA raw readings were then mapped to miRBase version 22 using two web-based tools Chimira and sRNAbench [31–33]. Both Chimira and sRNAbench read counts were normalized and analyzed using Bioconductor R package EdgeR with a deciding criterion |fold change|> 1.5 and FDR < 0.1 to identify differentially expressed miRNAs [34]. The DE miRNAs obtained from two mapping tools were then merged for a more inclusive outcome.

#### miRNA Target Mining

The predicted miRNA targets were obtained from miRWalk 2.0 by searching the miRNA binding sites in 5′-UTR, 3′-UTR, and CDS [35]. The predicted miRNA targets were then selected using the DE mRNAs as previously described [12]. Only the mRNA reversely expressed to the miRNA was selected as a target of interest.

#### Gene Ontology (GO) Enrichment Analysis

All the GO term enrichment analyses of the miRNA targets were conducted using an online tool named ShinyGO v0.66 [36].

#### miRNA GO-Term and mRNA Networking Visualization

The network visualization was done by using Cytoscape V3.8.2 [37].

#### Statistical Analysis

Statistical analyses were conducted using GraphPad Prism 9 software (GraphPad Software Inc., La Jolla, CA, USA). For comparisons, unpaired *t* tests were used to establish statistical significance. Data were expressed as the mean ± SD and written with the identification of *n* under appropriate figure legends, with *p < 0.05, **p < 0.01, ***p < 0.001, and ****p < 0.0001.

## Results

### α1ACT Induces miRNA Expression Profiles

To understand the role of miRNAs in α1ACT-regulated gene expression and cell differentiation, we used small RNA-seq to examine the simultaneous expression profiles of miRNAs in cells overexpressing α1ACT. miRNA reads were made from the same samples used for mRNA extraction in our previous at four different time points 6 h, 24 h, 3 days, and 10 days, with growth stages ranging from starting log phase to differentiating and senescent cell, post-plating of neural-crest-derived pheochromocytoma (pc12) cell lines, stably expressing pcDNA3 vector (pc12_pcDNA3_), and α1ACT (pc12_α1ACT_) as described previously [12, 21, 25, 26]. Using two mapping tools, Chimira and sRNAbench, the small RNA-seq obtained an average of 3 million mapped reads and 2.7 million miRNA reads per sample, with an average miRNA mapping rate of 88.9%. Out of a total of 764 miRNAs present in rat miRBase version 22, 596 miRNAs were detected in at least in one of 12 sequenced groups.

**Fig. 1:**
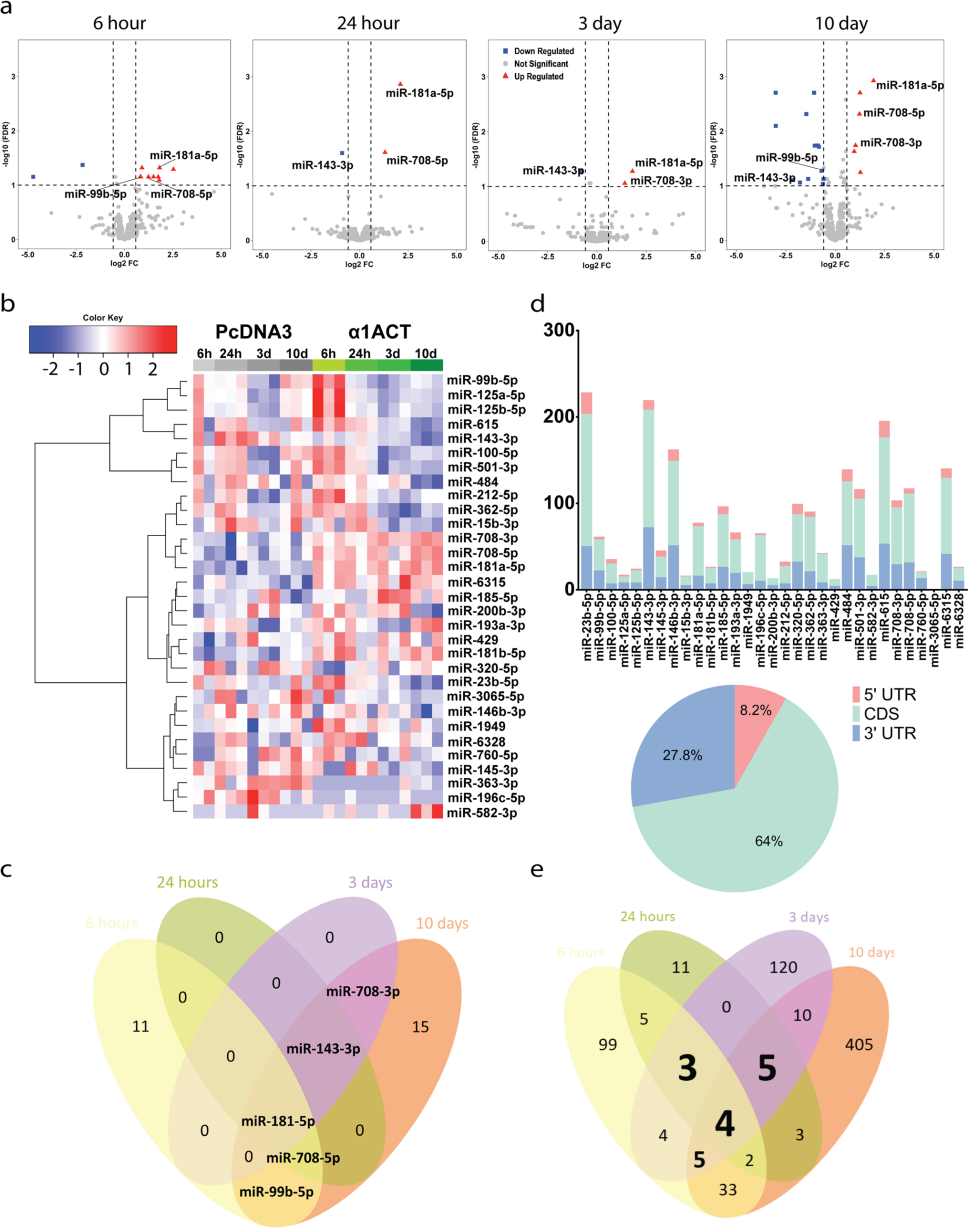
Differentially expressed miRNAs (DEMs) associated with α1ACT overexpression. **a** Volcano plot of DEMs between pc12 cell lines stably expressing α1ACT compared to EV at 4 different time points at |FC|> 1.5 and FDR < 0.1. Upregulated genes are marked in light red, and downregulated genes are marked in light blue. **b** Heat map of log2-normalized CPM expression values of 31 DEMs apparently regulated by α1ACT at 4 different time points in biological replicates of pc12 cell lines expressing α1ACT and EV. **c** Venn diagrams of potential putative DEMs associated with α1ACT at 6 h, 24 h, 3 days, and 10 days. miR-181a-5p was one of consistent DEMs in pc12 cell lines stably expressing α1ACT compared to EV cross the all 4 time points. **d** Distribution of DEMs binding events on 3′-UTR, CDS, and 5′-UTR targeting previously reported DEGs. **e** Venn diagram showing the overlaps of the DEGs previously reported that are predicted as miRNA targets at the different time points

Differentially expressed miRNAs (DEMs) were identified with the intersection of two criteria: (a) a false detection rate (FDR) of less than 0.1 using an R Bioconductor package edgeR and (b) fold change of more than 1.5, that is, miRNAs that showed more than 1.5-fold difference when compared to both controls (Fig. 1a and b). Thirty-one unique DEMs were identified as shown in the Venn diagram (Fig. 1c), 16 upregulated and 15 downregulated. Among these, 5 miRNAs were differentially expressed at least at two different time points. miRNA-181a was significantly upregulated through all time points; miRNA-708-5p was upregulated at 3 time points; miRNA-708-3p was upregulated at 2 time points; miRNA-143-3p was downregulated at 3 time points; miRNA-99b-5p was changed at 2 time points. Interestingly, unlike in other miRNA population studies, α1ACT overexpressing cells displayed similar proportions of both downregulated 2.7% (16/596) and upregulated 2.5% (15/596) DEMs. The downregulated DEMs included miR-15b-3p, 23b-5p, 320-5p, 143-3p, 146-3p, 362-5p, 363-3p, 484, and 615. The upregulated DEMs include several members of the mIR-708a/b, mIR-125a/b miR-181, 185, 193, 212, 582, and 6315. The expression of miR-99-5p and miR-125ab were upregulated at early time points but decreased at later time points. miRNAs are often present as clusters in the genome, transcribed together from physically adjacent miRNAs, and show similar expression profiles. Since a single miRNA can target multiple genes and miRNA clusters contain multiple miRNAs, it is important to understand their regulation, effects, and various biological functions [38, 39]. Among 31 DEMs, five miRNA clusters were detected in our study, including miR99/100/125, miR200b/429, miR143/145, miR181a/181b, and miR362/501 clusters.

The perfect binding between the seed region (5′ 2–8 nucleotides 3′) of mature miRNA and 3′-UTR of their mRNA target by Watson–Crick base pairing is considered to be the major determinant in blocking the target mRNA either by translational repression or mRNA degradation [40]. Although miRNA binding sites are most common in 3′-UTRs of mRNAs, there are some reports of miRNA interaction within the 5′-UTR, mRNA coding region, and intron–exon junctions [41, 42]. In our study, we found that the distribution of DEM binding events on 3′-UTR, CDS, and 5′-UTR targeting previously reported DEGs were 27.8%, 64%, and 8.2%, respectively (Fig. 1d).

### Transcription Factor α1ACT Indirectly Regulates mRNA Expression Profiles

As we reported, α1ACT is a transcription factor that orchestrates a network of pathways to mediate morphological and functional maturation of PCs and their connections. The integrated RNA-seq/ChIP-seq analysis revealed that α1ACT regulates a large ensemble of developmentally controlled genes, near 50% of which are directly involved in the development of different cerebellar compartments through direct binding [12]. The integrated ChIP-Seq and RNA-Seq data revealed that 52–67% of RNA-Seq DEGs were identified in the ChIP-Seq as bona fide gene targets of α1ACT which indicates that the rest of RNA-Seq DEGs, 424 out of 1272, are indirectly regulated by α1ACT. Two thirds of these are upregulated, and one third of DEGs are downregulated. We hypothesized that a portion of the DEGs *lacking α1ACT interaction by ChIP-seq* might be regulated at the post-transcriptional level through miRNAs.

### α1ACT Modulates the Paired Expression Profiles of miRNA and mRNA Target Relationship

**Fig. 2:**
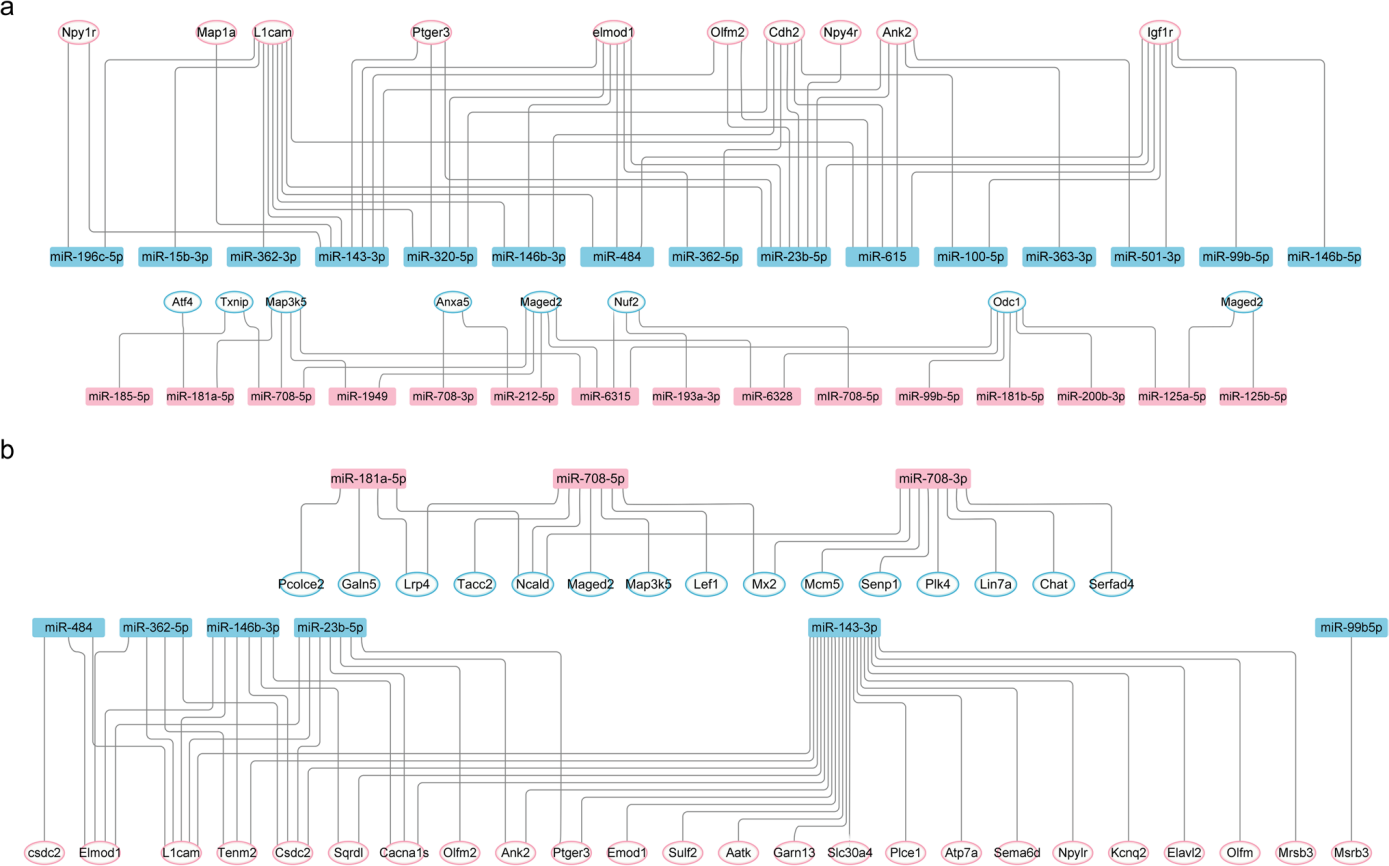
miRNA target mining. **a** Ten upregulated DEGs and 8 downregulated DEGs predicted as miRNA targets and regulated by multiply DEMs. Upregulated miRNAs are labeled in warm color, while downregulated miRNAs are labeled in cold color. **b** Three upregulated DEMs and 6 downregulated DEMs predicted target multiple DEGs. Upregulated miRNAs are labeled in warm color, while downregulated miRNAs are labeled in cold color

The identification of putative mRNA targets may help reveal the biological role of the differentially regulated miRNAs. For systematic miRNA target prediction, we analyzed the same set of RNA-seq and small RNA-seq data to monitor the expression of both miRNAs and candidate targets [43], using two different approaches for target identification. In the first approach, we employed a comprehensive miRNA target mining database miRWalk for target prediction, which ranks predicted miRNA target genes based on their anti-correlated expression behavior relative to their respective miRNA host genes. In the second approach, we input mRNA expression profiles from our previous study based on ChIP-seq-unrelated DEGs. Putative targets of DEMs are shown in Fig. 1e and File S1–4. Literature has suggested that a single miRNA can target several mRNAs together, and a single mRNA can be targeted by different miRNAs in a concerted manner [44]. Interestingly, 16 of 18 ChIP-seq-unrelated DEGs were targeted by DEMs. The relationship between one DEG targeted by two or more the DEMs was shown in Fig. 2a. L1cam, ELmod1, and tenm2 were targeted by 9, 7, and 6 downregulated DEMs, respectively (Fig. 2a). Co-targeting of individual mRNAs by different miRNAs could potentially achieve stronger and more complex patterns of repression. The relationship that one DEM targets two or more DEGs was shown in Fig. 2b. Clustering of all co-targeting pairs revealed a group of nine predominantly brain-enriched miRNAs that share many targets (Fig. 2b). Genes that are targets of multiple miRNAs are likely to be tightly regulated and may show graded response on the basis of expression of different miRNAs [45]. A large number of miRNAs and the capacity of one miRNA regulating several transcripts suggest a complex regulatory network to fine tune the gene expression and a mechanism by which they are thought to regulate various processes [44].

### α1ACT Expression Correlates with the Downregulation of the miR-99 Cluster and the Upregulation of Neurogenesis DEGs

**Fig. 3:**
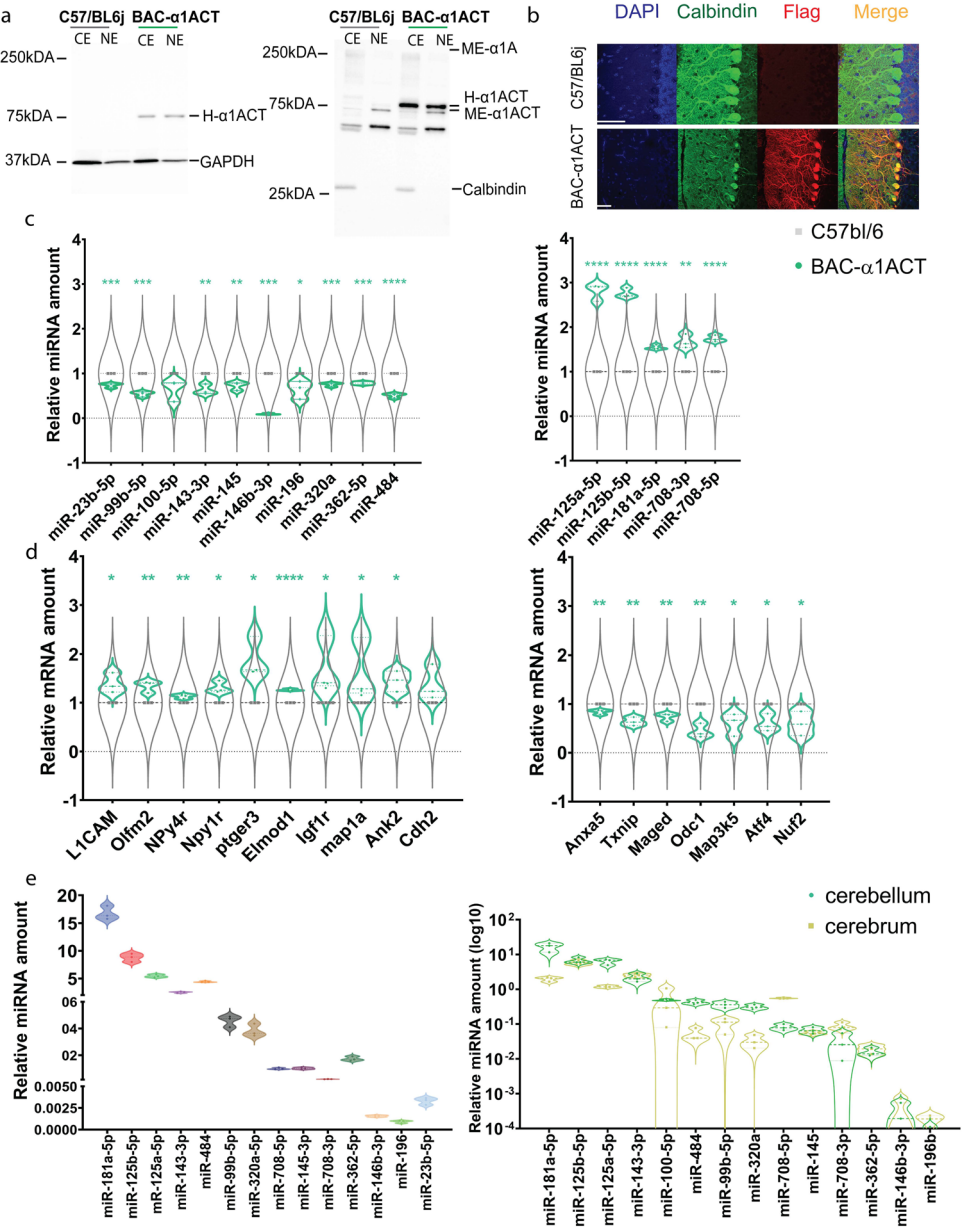
Five upregulated DEMs and 10 downregulated DEMs associated with α1ACT overexpression in mouse cerebellum. **a** The humanized α1ACT expression in nuclear and cytoplasm fractions of mouse cerebellum detected by anti-flag antibody (**a** left) and anti-α1ACT antibody(CT2) (**b** right). **b** The immunofluorescence staining of BAC-α1ACT mouse cerebellum. α1ACT expression in cerebellar Purkinje cells. Calbindin is in green and flag is in red. Scale bar represents 20 µm. **c** Fifteen of 33 DEMs were verified in α1ACT overexpression mouse cerebellum. Among them, 10 DEMs were downregulated (left), and 5 DEMs are upregulated (right). Comparison of miRNA expression level between BAC-α1ACT and C57/bl6j mice cerebellum at P22 (mouse *n* = 7, *n* = 8, respectively; experiment *n* = 3, *p < 0.05) using miR-186 as endogenous control. **d** Seventeen DEGs were verified in α1ACT overexpression mouse cerebellum. Among them, 10 DEGs are upregulated (left), and 7 DEGs were downregulated (right). Comparison of mRNA expression level between BAC-α1ACT and C57/bl6j mice cerebellum at P22 (mouse *n* = 7, *n* = 8, respectively; experiment *n* = 3, *p < 0.05) using beta-actin as endogenous control. **e** miR181 enriched in neonatal C57/bl6j mouse cerebellum using miR-186 as endogenous control (right, mouse *n* = 8; experiment *n* = 3). miRNA-181 is 16 times more enriched in the neonatal cerebellum than the brain (left, mouse *n* = 3; experiment *n* = 3)

In our previous study, the 5 of 18 ChIP-seq-unrelated common DEGs, L1cam, Elmod1, Ptger3, Npy4r, and Olfm2 were verified to be increased in α1ACT-transgenic mice (Fig. 3a and b) [21]. These genes are known to promote neurite outgrowth and neurogenesis. In this study, the ChIP-seq-unrelated DEGs, which were detected at two more time points of RNA-seq and were predicted targets of DEMs (mRNA-paired DEMs), were verified by Taqman expression assay in humanized α1ACT transgenic mice. Npy4ir, Igf1r, and Map1a were verified and increased at 1.13 ± 0.04, 1.77 ± 0.56, and 1.67 ± 0.61fold (Fig. 3d left). The mRNA-paired DEMs, miR-23b-5p, miR-99b-5p, miR143-3p, miR146b-3p, miR-362-5p, and miR-484, were validated using the cerebellar miRNA/mRNA co-extraction from the α1ACT transgenic mice by Taqman expression assay. These DEMs are decreased to 73.659% ± 5.160%, 54.071% ± 5.961%, 63.316% ± 11.453%, 9.403% ± 1.454%, 78.235% ± 3.394%, and 50.716% ± 5.318%, respectively, in α1ACT overexpression mice compared to those in C57BL/J mice (Fig. 3c left). These observations are consistent with the view that downregulation of these DEMS by α1ACT may reverse the suppression of the neurogenesis gene clusters and facilitate accelerated cell differentiation and neurogenesis.

### α1ACT Expression Correlates with the Upregulation of miR181 and miR-708 Cluster and Inhibition of MAPK Signaling and Cell Death Pathways

Three of 11 upregulated DEMs, miR-181a-5p, and the miR-708-3p/5p, were verified and increased in α1ACT-transgenic mice, 1.54 ± 0.05, 1.67 ± 0.15, and 1.74 ± 0.07-fold, respectively (Fig. 3c right). The Taqman gene expression assay for miR-181a-5p and miR-708-3p/5p target DEGs was further performed. We found that Odc1, Ask1, Atf4, and Nuf2 were decreased to 55.75% ± 14.50%, 40.23% ± 23.19%, 39.99% ± 18.14%, and 40.35% ± 24.80% in α1ACT-transgenic mice (Fig. 3d right). Ask1 is a key factor in mitogen-activated protein kinase (MAPK) signaling pathway and an upstream activator of the c-Jun N-terminal kinase (JNK) and p38 MAPK signaling cascades to relay death signals into cells [46]. ASK1 is also implicated in neuronal differentiation [47]. Previous studies have found that depletion of ASK1 by RNA interference modulates neurite outgrowth [48]. In addition, other studies have revealed that Odc1, Atf4, and Nuf2 are involved in cell cycle arrest [49].

### α1ACT-Associated DEMs Are Enriched in the Cerebellum

Seventy percent of all known miRNAs are expressed in the brain, owing in part to the diverse types of neurons and non-neuronal cells [50]. Surprisingly, only a handful of microRNAs are expressed in a brain-specific or brain-enriched manner [51].

To investigate the enrichment of α1ACT-associated DEMs in neonatal cerebellum, we compared the expression levels of DEMs between cerebellum lysates and brain lysates in α1ACT overexpression mice at p20. MiR-181b-5p, miR125a/b-5p, miR-143-3p, miR-100-5p, miR-484, and miR-99-5p were the top 7 α1ACT-associated DEMs in cerebellum which were enriched 16.87 ± 4.82, 6.34 ± 1.22, 6.76 ± 1.35, 2.17 ± 0.62, 0.49 ± 0.026, 0.43 ± 0.062, and 0.36 ± 0.07-fold (Fig. 3e left). Importantly, we provide the first evidence that the cortex-enriched miRNA-181, which was induced during neuronal differentiation, is 16 times more enriched in neonatal cerebellum than cerebrum (Fig. 3e right).

### Functional Annotation Analysis of the miRNA/mRNA Network

**Fig. 4:**
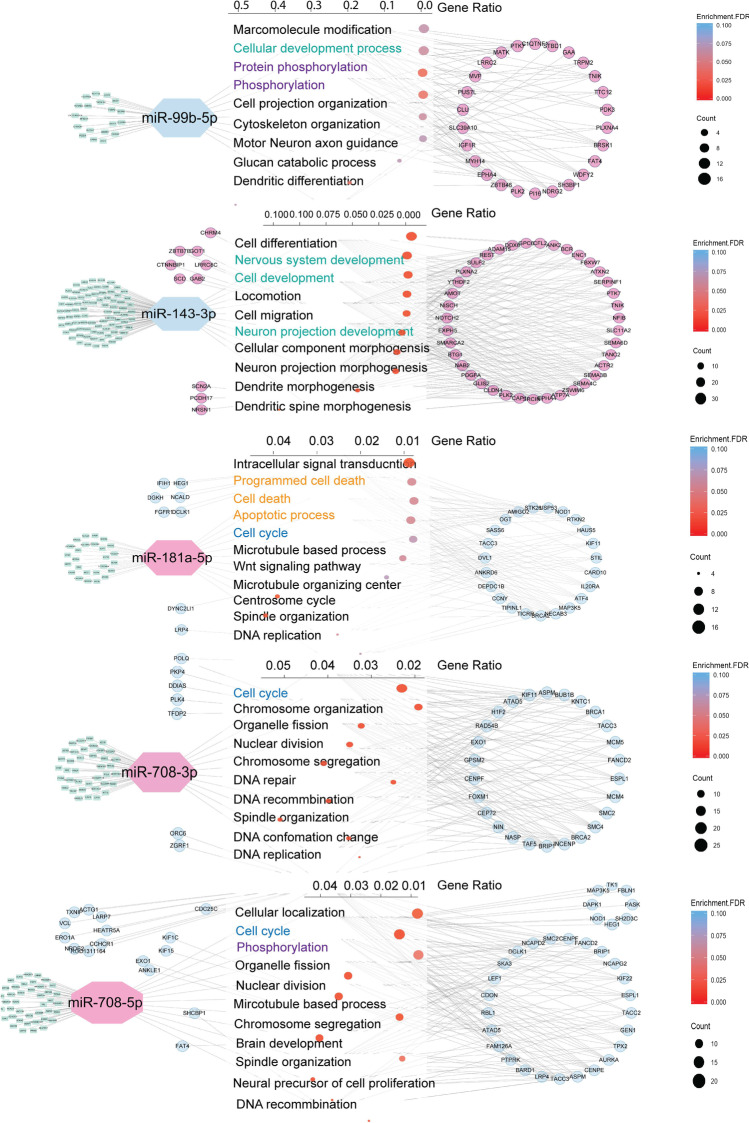
Functional annotation and miRNA/mRNA networking. Dot plot of the top 10 selected biological process GO term of each miRNA. Upregulated miRNA and mRNA are labeled in warm color, while downregulated ones are labeled in cold color

To systemically explore the biological function associated with the DEMs/DEGs pairs modulated by α1ACT, we applied functional enrichment analysis on the miRNA target DEGs. We used ShinyGo V0.66 [36] to conduct the Gene Ontology enrichment (GO term) analysis using Enrichment FDR < 0.1 as criteria. The downregulated DEMs, miR-23b-5p, miR-99b-5p, miR143-3p, miR146b-3p, miR-362-5p, and miR-484, target to the verified DEGs, L1cam, ELmod1, Olfm2, Npy4r, Ptger3, and Cdh2 that were increased after α1ACT overexpression. The top 5 GO terms of these DEGs were related to nervous system development and neuron morphogenesis. In this case, under α1ACT overexpression, the downregulation of these DEMs eliminates the repression for the neurogenesis-related pathways to promote neuronal development. The DEGs targeted by 3 verified upregulated DEMs, miR-181a-5p, and the miR-708-3p/5p were downregulated by α1ACT overexpression. The top 5 GO terms of these DEGs are involved in cell death, cell cycle, and DNA repair. With α1ACT overexpression, DEMs were upregulated, which leads to suppression of the respective DEGs which might further inhibit the cell death and MAP pathway. The expression profiles regulated indirectly by α1ACT appeared to act through miRNAs to both enhance neurogenesis and oppose cell death (Fig. 4).

## Discussion

Here, we used an integrated analysis of mRNA and miRNA expression profiles to study the effect of α1ACT on the miRNA expression profile to infer a role for miRNA networks in the indirect control of gene expression by α1ACT during cerebellar development. We sequenced mRNAs and small RNAs and verified their expression from the identical extraction of α1ACT-overexpressing mouse cerebellum. This approach provides a more accurate assessment of transcriptome changes on the same scale. miRNA and mRNA co-sequencing at α1ACT overexpression transcriptome opens opportunities to investigate how changes in RNA expression modulated by α1ACT might contribute to neurogenesis and synaptic formation and how this coordinated activity might play a prominent role in controlling neonatal cerebellar development. The results of this study provide further insight that α1ACT, through indirect regulation, controls the fate of cerebellar neuronal homeostasis during neonatal development by balancing the processes of neurogenesis and cell death.

Over the first 9 months of life, the weight of cerebellum increases from 5.7 to 10% of the total brain weight. Cerebellar neuronal cells possess the ability to proliferate in a short time window during fetal and early postnatal life. Numerous studies demonstrate that miRNAs function at all stages of neuronal development, ranging from the initial specification of neuronal cell types to the formation, morphological dynamic modifications, and plasticity of synaptic connections between individual neurons [20, 51–54]. There are 16 downregulated DEMs identified regulated by α1ACT in our study. miR-23b-5p, miR-99b-5p, miR-143-3p, miR-146-3p, miR-362-5p, and miR-484 were verified as increased in the neonatal cerebellum of α1ACT-overexpressing mouse. All of these miRNAs paired with the previously verified upregulated DEGs involved in neurogenesis, neuronal morphological, and synaptic plasticity [12]. This provides evidence that miR-23b-5p, miR-99b-5p, miR-143-3p, miR-146-3p, miR-362-5p, and miR-484 play essential roles in controlling cerebellar neurogenesis and migration through α1ACT activation.

Two parallel, intrinsically linked processes occur during development of the nervous system: the generation of new neurons and glia and the death of cells that are no longer required and/or are produced in excess [55–57]. In the neonatal cerebellum, neuronal differentiation and proliferation are active as the rate of cell death decreases [58]. One way in which this connection may be achieved is through the coupling of the neurogenetic process and cell cycle, perhaps by controlling a shared set of factors [59]. In our study,15 DEMs appear to be upregulated by α1ACT: miR-181a-5p, miR-708-5p/-3p, miR-146-3p, miR-362-5p, and miR-484 were verified in the cerebellum of the α1ACT-overexpressing mouse. The DEGs paired with these DEMs are involved in cell death and cell cycle arrest including genes Ask1, Odc1, Atf4, and Nuf2 that were repressed in the cerebellum during the neonatal period. In this manner, α1ACT may represent one of the shared factors, facilitating an increase in cell proliferation with the downregulated miRNAs and an upregulation of neurogenesis genes as a neuroprotective strategy to regulate cell death in cerebellum. α1ACT plays a vital role in the homeostatically balanced process of cell loss and cell gain to generate and maintain the complex architecture of cerebellum and to allow adaptation to changing circumstances after birth.

Taking into account the fact that each miRNA can regulate, on average, the expression of 100–200 target genes [60, 61] and transcriptome-wide correlation of the miRNA with mRNA expression profiles with α1ACT overexpression revealed a set of target genes showing an inverse relationship with miRNA levels. In our study, 31 DEMs and 2,365 DEMs/DEGs pairs were identified modulated by α1ACT. Most of them are multi-pairing. Those DEMs and DEGs involved in pairs contribute to neurogenesis, morphologic and synaptic formation, and cell death controlling neonatal cerebellar development. α1ACT seems to utilize the miRNA/mRNA network to regulate a group of genes in neuronal genetic process and multiple cross-talk pathways in cell death and cell cycle, which may have a significant impact on the cerebellar developmental regulatory network and ultimately the physiological processes of cerebellum.

Interestingly, two of three upregulated DEMs, miR-181a-5p and miR-708-5p both targeted to Ask1 gene. Phosphorylated Ask1, as a key member of the MAPK signaling pathway, induces activation of JNK and p38 MAPK. In turn, activated JNK and p38 MAPK regulate autophagy, thereby modulating apoptosis, proliferation, and maintaining cellular integrity [46, 62]. Ask1 participates in diverse biological pathological processes, such as cell death, survival, and differentiation. It has been suggested as a therapeutic target in various diseases [63]. Accumulating evidence indicates that Ask1 plays a key role in the pathogenesis of neurodegenerative diseases (NDDs), such as Alzheimer’s disease, Parkinson’s disease, and Huntington’s disease [64]. Meanwhile, depletion of Ask1 blunts autophagy and stabilizes the survival neuron protein [48], suggesting it may be a new point of therapeutic intervention to prevent or treat NDDs[65].

miRNAs potentially are organized into related clusters. These clusters target multiple mRNA transcripts within common cellular pathways including proliferation, differentiation, and apoptosis. A total of five clusters, miR99/100/125, miR200b/429, miR143/145, miR181a/181b, and miR362/501 clusters, were identified in our study. The miR143/145, miR200b/429, and miR362/501 clusters were downregulated, stimulating neurogenesis, migration, and synaptic formation. The miR181a/181b cluster was upregulated, leading to suppression of the neuronal cell death pathway. Further, network analysis reveals that these miRNA clusters have a capacity to coordinately regulate multiple steps within a pathway, providing a dynamic and complex control of entire pathways. We also identified several miRNAs were highly expressed in neonatal cerebellum including miR181a/181b cluster. An increasing variety of miRNAs being identified in the cerebellum suggests a complex connection between dynamics of action and regulatory potential of miRNAs in the cerebellum.

In summary, this study discovered that miRNAs mediated by α1ACT facilitate the inverse relationship between neurogenic processes and cell death during neonatal cerebellar development. Combined with our previous findings, these studies merge the whole gene expression profiles regulated both directly and indirectly by α1ACT and reveal a unique role for α1ACT in maintaining neuronal homeostasis. In broader terms, it has recently been shown that cell turnover, including neurons, does occur in the mature central nervous system. The persistence of precursor cells that possess the functional characteristics of bona fide neural stem cells within restricted brain areas [66] provides the potential neurogenetic opportunity to treat the neurodegenerative disease in adults. Since α1ACT is able to orchestrate the ensemble of genes through the direct and indirect regulation and balance of the neurogenetic and neurodegenerative process in neonates, it is feasible that α1ACT will be a unique candidate in therapeutic strategies for NDDs.

## Supplementary Information

Below is the link to the electronic supplementary material.

Supplementary file1 (CSV 136 KB)

Supplementary file2 (CSV 299 KB)

Supplementary file3 (CSV 947 KB)

Supplementary file4 (CSV 38 KB)
