# Correction: The Transcription Factor, α1ACT, Acts Through a MicroRNA Network to Regulate Neurogenesis and Cell Death During Neonatal Cerebellar Development

**DOI:** 10.1007/s12311-023-01567-9

**Published:** 2023-05-18

**Authors:** Cenfu Wei, Kellie Benzow, Michael D. Koob, Christopher M. Gomez, Xiaofei Du

**Affiliations:** 1 Department of Neurology, University of Chicago, Chicago, IL 60637 USA; 2 Department of Laboratory Medicine and Pathology, University of Minnesota, Minneapolis, MN 55455 USA

**Correction to: The Cerebellum**

10.1007/s12311-022-01431-2

The article “The Transcription Factor, α1ACT, Acts Through a MicroRNA Network to Regulate Neurogenesis and Cell Death During Neonatal Cerebellar Development”, written by Cenfu Wei, Kellie Benzow, Michael D. Koob, Christopher M. Gomez and Xiaofei Du, was originally published electronically on the publisher’s internet portal on 22 June 2022 without open access. With the author(s)’ decision to opt for Open Choice the copyright of the article changed on 05 May 2022 to © The Author(s) 2023 and the article is forthwith distributed under a Creative Commons Attribution 4.0 International License, which permits use, sharing, adaptation, distribution and reproduction in any medium or format, as long as you give appropriate credit to the original author(s) and the source, provide a link to the Creative Commons licence, and indicate if changes were made. The images or other third party material in this article are included in the article’s Creative Commons licence, unless indicated otherwise in a credit line to the material. If material is not included in the article’s Creative Commons licence and your intended use is not permitted by statutory regulation or exceeds the permitted use, you will need to obtain permission directly from the copyright holder. To view a copy of this licence, visit http://creativecommons.org/licenses/by/4.0.

